# A metabolic switch for myelination

**DOI:** 10.1186/s13619-025-00277-3

**Published:** 2025-12-15

**Authors:** Shenghui Niu, Lin Zhao, Da Jia

**Affiliations:** Key Laboratory of Birth Defects and Related Diseases of Women and Children, Department of Paediatrics, West China Second University Hospital, State Key Laboratory of Biotherapy and Collaborative Innovation Center of Biotherapy, Sichuan University, Chengdu, 610041 China

## Background

**Fig. 1:**
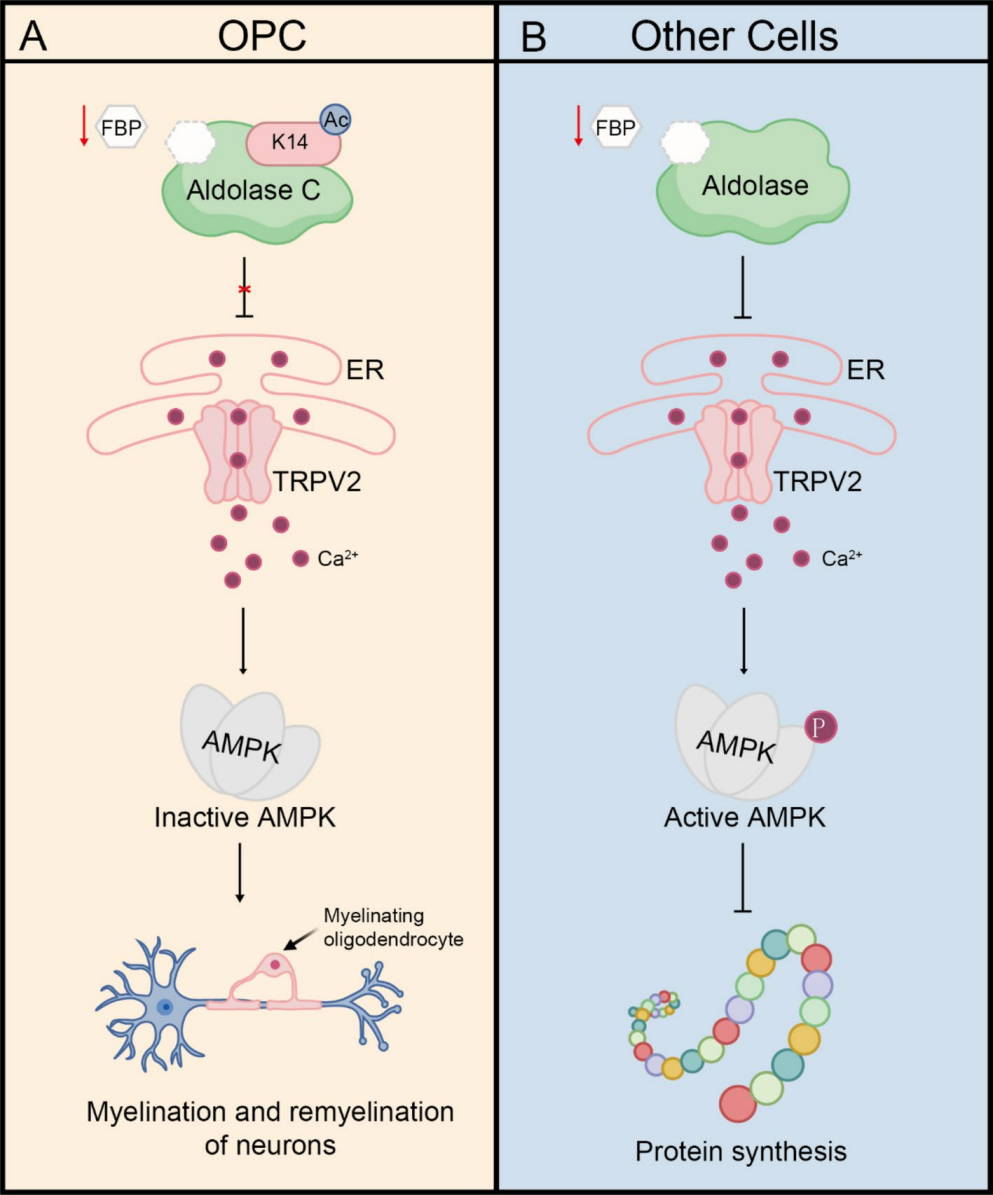
Post-translational modification of aldolase serves as a crucial molecular switch regulating its pivotal function. Sun et al. discovered that acetylation at the K14 site of aldolase C(ALDOC) in OPCs plays a checkpoint role in activating AMPK under low glucose conditions (right). It ensures normal myelin production by blocking rather than activating the lysosomal AMPK pathway. This is distinctly different from the response mechanism of other cells: in the latter, aldolase in an "idle" state senses the decrease in FBP under low glucose conditions and activates AMPK by inhibiting the TRPV channel, thereby disrupting anabolism and initiating catabolism to maintain homeostasis (left)

In an important advancement in the field of metabolic research, a study recently published by Sun et al. revealed a new mechanism by which cell types specifically regulate AMP-activated protein kinase (AMPK) activation in response to low-glucose stimulation. This study indicates that the acetylation of the glucose sensor aldolase C (ALDOC) in oligodendrocyte precursor cells (OPC) at the lysine 14 position serves as a key "metabolic checkpoint". The aldolase family proteins (ALDOA, ALDOB and ALDOC) usually sense the deficiency in glucose, activate the lysosomal AMPK pathway, and inhibit cell proliferation and differentiation. However, acetylation of ALDOC shields the cells from low-glucose signals, and permits the proliferation and differentiation of OPCs for myelination (Sun et al. 2025) (Fig. 1 left).

## Main text

AMPK is the core regulator of metabolic homeostasis and can sensitively respond to fluctuations in energy and nutrient supply (Lin and Hardie 2018; Steinberg and Hardie 2023; Trefts and Shaw 2021). Recent studies have identified that AMPK is the main sensor for glucose. At low glucose levels, aldolase proteins sense the decline in FBP levels and stay in an "idle" state. The "idle" aldolase interacts with endoplasmic reticulum (ER)-localized transient receptor potential channel V subfamily (TRPV) and inhibits its activity, thereby suppressing calcium release under low-glucose conditions. The subsequent decrease in calcium ion concentration at the endoplasmic reticulum-lysosomal contact site enables the inhibited TRPV to bind to lysosomal v-ATPase, thereby driving the activation of AMPK. Importantly, this glucose-sensing pathway is independent of AMP (Gonzalez et al. 2020; Li et al. 2019; Zhang et al. 2017; Zhang et al. 2014; Zong et al. 2019) (Fig. 1, right). However, in certain tissues, the anabolic and catabolic activities of different cell types are not synchronized, suggesting that the AMPK regulatory network exhibits pronounced cell-type specificity. The molecular basis behind these spatiotemporal regulation characteristics still awaits further revelation.

Sun et al. found that OPCs in the cerebral cortex exhibited a unique metabolic phenotype: even under low-glucose stress conditions, their AMPK signaling pathway remained inactive. Further research indicated that this obvious "insensitivity" to energy stress was not a passive defect but an active adaptation mechanism adopted to fulfill its key biological functions. The core mission of oligodendrocyte precursor cells is to proliferate and differentiate into mature oligodendrocytes (mOLs), which wrap axons by synthesizing myelin sheaths. This process requires a large amount of protein and lipid biosynthesis and is in a highly anabolic state. If oligodendrocyte precursor cells activate AMPK and inhibit anabolism during mild energy deficiency, it will disrupt the ongoing myelin formation and repair process, ultimately damaging neurological function. Thus, the metabolic "insensitivity" of OPCs to low glucose constitutes a physiological adaptation essential for sustaining effective myelination and efficient neural signal transmission.

Mechanically, Sun et al. revealed ALDOC, the predominant aldolase protein expressed in OPC, had undergone a crucial acetylation modification of its 14th lysine (K14). This modification acts like an "insulating switch", preventing aldolase from binding and inhibiting TRPV under low-sugar conditions, thereby blocking the activation signal of AMPK. Functional experiments confirmed that the expression of the ALDOC-K14R mutant mimicking the deacetylated state could restore the sensitivity of OPC to low glucose and activated AMPK, while its proliferation and differentiation abilities were significantly impaired. This directly proved that K14 acetylation was crucial for maintaining the function of OPC. Interestingly, when OPC differentiates into mOLs, the expression level of its deacetylase Sirtuin 2 (SIRT2) is significantly upregulated, thereby removing the acetyl group at the ALDOC K14 site. This change is equivalent to turning off the "insulating switch", enabling mOLs to sense and respond to fluctuations in glucose levels normally just like ordinary cells. This discovery clarified the ingenious strategy by which the same cell lineage dynamically regulates its metabolic perception ability through reversible acetylation modification at different differentiation stages.

Remyelination is one of the few regenerative processes in the central nervous system that rely on the proliferation and differentiation of endogenous OPCs into mOLs. The deficiency of OPCs is associated with a variety of demyelinating diseases. To explore the physiological significance of AMPK inactivation, Sun et al. constructed multiple demyelinating models and found that the glucose concentration in the demyelinating region decreased significantly while AMPK was not activated. In mice specifically expressing ALDOC-K14R in OPC, AMPK was abnormally activated in OPC, and its proliferation and differentiation were hindered, resulting in myelin regeneration defects and aggravated symptoms. Conversely, knockout of AMPKα1/α2 in this context could reverse the above defects, indicating that ALDOC-K14R inhibited myelin formation through AMPK. Sun et al. 's work revealed the crucial role of ALDOC acetylation as a metabolic checkpoint in maintaining OPC function and myelin integrity in a low-glucose environment. This discovery offered mechanistic insight and potential therapeutic targets for promoting remyelination, such as targeting ALDOC acetylation.

As a core regulatory hub of metabolic pathways, the post-translational modification of aldolase constitutes a key molecular switch mechanism. In liver cancer research, the β -hydroxybutyric modification (Kbhb) of the ALDOB protein Lys108 directly inhibited its enzymatic activity and binding to the substrate FBP, and thereby effectively inhibited the proliferation of cancer cells (Qin et al. 2024). Similarly, the crotonylation modification (Kcr) at Lys367 of aldolase TaFBA6 in wheat chloroplasts enhanced the structural stability of the protein by regulating the surface potential. This not only improved the activity and tolerance of the enzyme in alkaline environments but also directly enhanced the salt tolerance of plants (Zhu et al. 2023). These findings clearly revealed that chemical modifications at specific sites can directly and efficiently reconstruct the function of aldolase, achieving precise regulation of metabolic pathways.

Glucose is not only the main carbon source for most cells to maintain life activities, but its availability also determines the switching between anabolism and catabolism in cells. While activating AMPK through the glucose-sensing pathway, it usually inhibits mammalian target of rapamycin complex 1 (mTORC1). Researchers also observed in OPCs expressing the ALDOC-K14R mutant that the activity of mTORC1 was significantly inhibited in demyelinating disease models. This finding indicated that ALDOC acetylation was pivotal for preserving mTORC1 activity and thereby supporting the anabolic programs that drive OPC proliferation, differentiation, and myelination. Nonetheless, the precise mechanism through which ALDOC acetylation modulated mTORC1 activity remained unclear.

Like many pioneering studies, the research by Sun et al. also raised several new scientific questions. First of all, at the molecular level, what is the precise structural basis for inhibiting TRPV channels by ALDOC acetylation? Is ALDOC function cross-regulated by modifications other than acetylation (such as phosphorylation, succinylation)? Secondly, at the physiological and pathological level, what is the cause of local glucose reduction in demyelinating lesions, and is this a driver or a consequence of pathology? Is this ALDOC-acetylation-dependent metabolic insulation unique to OPCs, or is it a broader regulatory principle employed by other highly anabolic cells, such as those in developing tissues or regenerative contexts? Finally, as the core of cellular metabolism, the upstream regulatory factors of the AMPK and mTOR pathways may be far more complex than what is known. Exploring other metabolic signaling substances other than glucose and amino acid deficiency (Mao et al. 2025) will provide us with a more complete map of collective energy metabolism regulation.

## Data Availability

Not applicable.
